# The longevity of *tor1Δ*, *sch9Δ*, and *ras2Δ* mutants depends on actin dynamics in *Saccharomyces cerevisiae*

**DOI:** 10.1186/s13578-015-0008-z

**Published:** 2015-04-18

**Authors:** Ying Liu, Nanqi Liu, Didi Wu, Qiang Bi, Shengnan Meng

**Affiliations:** Department of Biochemistry and Molecular Biology, Key Laboratory of Cell Biology, Ministry of Public Health, China Medical University, Shenyang, 110001, P. R. China; Department of Pharmaceutics, Pharmacy school of China Medical University, Shenyang, 110001 P. R. China

**Keywords:** Tor1, Sch9, Ras2, Longevity, Actin dynamic

## Abstract

Recent studies have revealed the role of actin dynamics in the regulation of yeast aging. Although the target of rapamycin (TOR) complex, serine/threonine kinase Sch9, and Ras2 have been shown to play important roles in aging for a long time, the relationship between these regulators and actin has not yet been reported. In this study we investigated the roles of actin polarization in *tor1Δ*, *sch9Δ*, *and ras2Δ* mutant cells. We found that the actin structures in *tor1Δ*, *sch9Δ*, and *ras2Δ* mutant cells were more dynamic than those in the wild type. Destruction of the actin structures with jasplakinolide decreased the life span of *tor1Δ*, *sch9Δ*, and *ras2Δ* mutants. Furthermore, deletion of *SLA1* in *tor1Δ*, *sch9Δ*, and *ras2Δ* mutants inhibited the actin dynamics and life span. In addition, we found that the actin cytoskeleton of the long-lived mutant *sch9Δ*, depended on the transcription factors RIM15 and MSN2/4, but not GIS1, while the actin skeleton of the *tor1Δ* and *ras2Δ* mutants depended on RIM15 as expected. Our data suggest that the longevity of *tor1Δ*, *sch9Δ*, and *ras2Δ* mutants is dependent on actin dynamics.

## Introduction

The budding yeast, *Saccharomyces cerevisiae*, has been successfully established as a model to study the mechanisms of aging. Currently, there are two models to study aging in yeast: the replicative life span (RLS), which represents the replicative capacity of a mother cell and is measured by counting the number of daughter cells produced by a mother cell [1,2], and the chronological life span (CLS), which is defined as the length of time the mother cell can survive in liquid medium [3,2].

Tor1 is a PIK-related protein kinase and one of the subunits of TOR complex 1 (TORC1). The TOR complex controls growth in response to nutrients by regulating translation, transcription, ribosome biogenesis, nutrient transport, and autophagy, which are important cellular responses implicated in increased life span [4]. Previous studies suggest that the TOR pathway controls mitochondrial respiration and production of reactive oxygen species (ROS) in yeast, which is thought to be an important determinant for life span in yeast cells [5-7].

The yeast kinase Sch9 can mediate glucose-dependent signaling, stimulate growth and cellular proliferation, and decrease stress resistance [8,9]. The COOH-terminal region of Sch9 is highly homologous to the AGC family members of serine/threonine kinases, which include Akt/PKB and S6K in mammalian cells. Sch9 can be phosphorylated directly by TOR1 at multiple sites and by PDK1 in the activation loop [10]. Major scientific discoveries into the regulation of longevity by Sch9 in yeast comes from Longo’s Lab [11]. They have shown that *sch9Δ* cells can survive three times longer than wild type cells. Stress-resistance transcription factors, such as Msn2/4 and protein kinase, Rim15, are required for this life span extension [12].

*RAS2* is a homolog of the mammalian oncogene, RAS, and is highly related to the *RAS1* gene in budding yeast [3]. It codes for a small GTP-binding protein and has been shown to regulate the nitrogen starvation response through its effects on adenylate cyclase (encoded by the *CYR1* gene). The GTP-bound form, Ras2, directly induces the production of cAMP by adenylate cyclase [10].

Previous studies have shown that actin dynamics regulates longevity though its underlying mechanism is still unclear [13,14]. It was suggested that decreased actin dynamics led to depolarization of the mitochondria and induced production of reactive oxygen species (ROS), which reduced cell longevity. Tor1, Sch9 and Ras2 are important regulators of aging, however, whether actin dynamics is required for longevity of *tor1Δ*, *sch9Δ*, and *ras2Δ* mutants is unknown.

In this study, the role of actin dynamics in the life span of *tor1Δ*, *sch9Δ*, and *ras2Δ* mutant yeast cells was investigated. Using genetic and biochemistry assays, we found that the actin pattern is more dynamic in *tor1Δ*, *sch9Δ*, and *ras2Δ* mutant cells than that in wild type cells, and that reduced actin dynamics, through either the actin stabilizing drug, jasplakinolide, or *SLA1* deletion, led to shorter life spans in these mutants. Although actin dynamics is required for all three mutants, the actin cytoskeleton in the long-lived mutant *sch9Δ*, depended on the transcription factors, RIM15 and MSN2/4, while *tor1Δ* and *ras2Δ* depended on RIM15. These data suggest that the regulation of actin dynamics in *tor1Δ*, *sch9Δ*, and *ras2Δ* is based on different mechanisms.

## Results

### The actin structure of *tor1Δ*, *sch9Δ*, and *ras2Δ* mutants is more dynamic than that of wild type cells

In order to test if the *tor1Δ*, *sch9Δ*, and *ras2Δ* strains used in this study have similar extended life spans as in the previous study, the same medium was used to examine the chronological life span as noted in the previous study [11]. As expected, *tor1Δ*, *sch9Δ*, and *ras2Δ* was smaller in size than the DBY746 wild type cells (data not shown). At day 8 after inoculation, the viability of DBY746 was less than 5%. The viability of *tor1Δ*, *sch9Δ*, and *ras2Δ* cells at the same time points, however, was 73%, 85%, and 82%, respectively. At day 11, almost all the wild type cells were dead, but the viability of *tor1Δ*, *sch9Δ*, and *ras2Δ* cells was 38%,78%, and 73%, respectively. These data suggest that the yeast mutants *tor1Δ*, *sch9Δ*, and *ras2Δ* have longer life spans, as reported previously [12].

**Figure 1:**
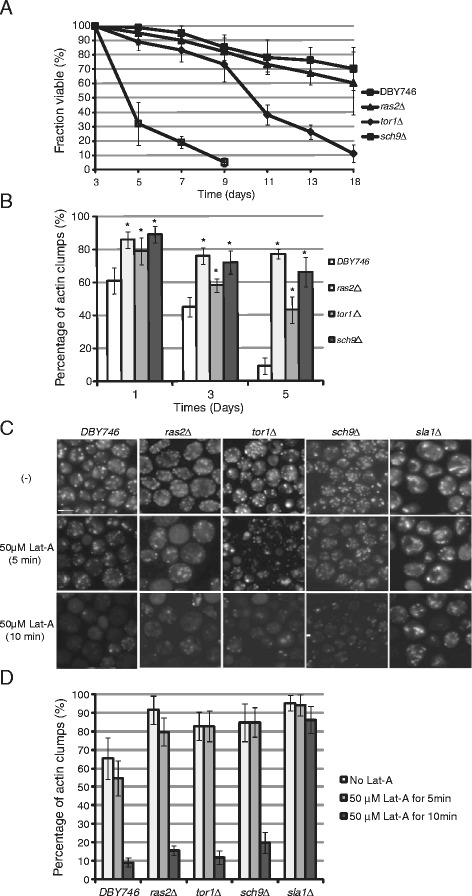
The actin structure is more dynamic in longevity mutants. **(A)** Wild type (DBY746), *tor1Δ* (LY101), *sch9Δ* (LY102), and *ras2Δ* (LY103) cells were cultured in aging medium. Triplicate cultures for each strain were used and experiments were repeated three times. Error bars represent standard errors. **(B)** The actin dynamic pattern was examined on day 1, 3, and 5 for yeast strains in **(A)**. *,p < 0.01. **(C)** Actin is dynamic in DBY746, *tor1*, *sch9Δ*, and *ras2Δ*. The cells were treated with 50 *μ*M latrunculin A, and after 5 and 10 min the actin pattern was examined by fluorescent microscopy. Scale bar, 5 *μ*m. **(D)** The actin dynamic pattern was quantified for yeast strains in **(C)**.

It was reported that increasing actin dynamics led to a longer life span [13]. Although a lot of studies have shown that Tor1, Sch9, and Ras2 are required for longevity, the actin dynamics in *tor1Δ*, *sch9Δ*, and *ras2Δ* cells were not examined. The dynamic actin structures in the wild type and mutant cells in Figure 1A were quantified. As shown in Figure 1B, the percentage of actin clumps in wild type cells decreased faster than that of *tor1Δ*, *sch9Δ*, and *ras2Δ* cells. On day 1, the percentage of actin clumps was about 62%, while in the *tor1Δ*, *sch9Δ*, and *ras2Δ* mutants, it is more than 78%. On day 5, the percentage of actin clumps is about 8%, while in *tor1Δ*, *sch9Δ*, and *ras2Δ* mutants, it is more than 42%. These results suggest that *tor1Δ*, *sch9Δ*, and *ras2Δ* have more dynamic actin structures compared to wild type cells. A previous study showed that cells with poor actin dynamics were resistant to the actin disrupting drug latrunculin-A (Lat-A) [15]. To test whether the actin structures in *tor1Δ*, *sch9Δ*, and *ras2Δ* are dynamic, we treated the wild type and *tor1Δ*, *sch9Δ*, and *ras2Δ* mutants with Lat-A, which destroys the actin structures; *sla1Δ* cells were used as the negative control. After 5 min of 50 *μ*M of Lat-A treatment, the percentage of cells with actin patches in wild type, *tor1∆*, *ras2∆*, *sch9∆* and *sla1∆* is 54.7%, 79.6%, 82.8%, 84.8%, and 94.2%, respectively. After 10 min of 50 *μ*M of Lat-A treatment, however, the percentage of cells with actin patches in wild type, *ras2∆, tor1∆*, *sch9∆* and *sla1∆* is 9.8%, 16.3%, 12.3%, 20.1%, and 85.5%, respectively. These data suggest that the actin structures in longevity cells are dynamic.

### Jasplakinolide treatment decreases the actin dynamics and life span of *tor1Δ*, *sch9Δ*, and *ras2Δ* mutants

**Figure 2:**
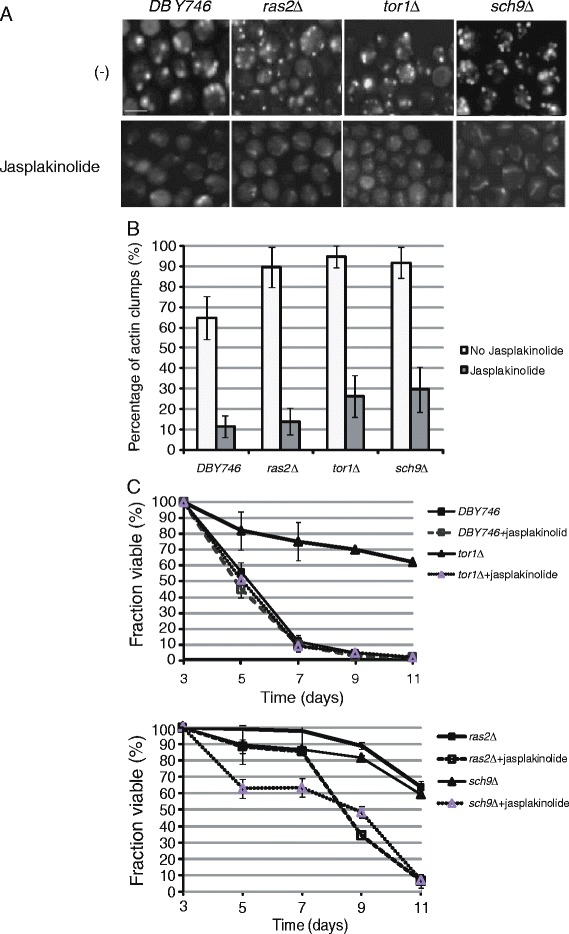
Dysfunction of actin dynamics after jasplakinolide treatment leads to reduced actin dynamics and reduced chronological life span in DBY746, *tor1Δ*, *sch9Δ*, and *ras2Δ* yeast cells. **(A)** 2 *μ*M of jasplakinolide was added into the medium. The actin dynamic pattern was examined by fluorescent microscopy. Scale bar, 5 *μ*m. Cells were fixed for 1 h by adding 37% formaldehyde solution directly to growth medium to a final concentration of 3.7%. After washing with 1× PBS solution for three times, cells were stained with phalloidin-TRITC in the dark at room temperature for 1 h and images were taken by fluorescent microscope. **(B)** The actin dynamic pattern was quantified for yeast strains in **(A)**. **(C)** Chronological life span in DBY746, *tor1Δ* (LY101), *sch9Δ* (LY102), and *ras2Δ* (LY103) cells decreased after jasplakinolide treatment.

Jasplakinolide is an actin-stabilizing chemical and can block actin dynamics [16]. To further test whether actin dynamics is involved in the longevity of *tor1Δ, sch9Δ*, and *ras2Δ* mutants, the wild type, *tor1Δ, sch9Δ*, and *ras2Δ* mutant cells were treated with 2 *μ*M jasplakinolide. As shown in Figure 2A and B, under the normal growth condition, the percentage of cells with actin patches in wild type, *ras2∆*, *tor1∆*, *sch9∆* and *sla1∆* is 64.7%, 89.6%, 94.8%, and 91.8%, respectively. After jasplakinolide treatment, however, the percentage of cells with actin patches in wild type, *ras2∆, tor1∆* and *sch9∆* is 12.5%, 14.7%, 27.2%, and 30.6%, respectively. These data suggest that the actin filaments was stabilized in wild type, *tor1∆*, *sch9∆*, and *ras2∆* after jasplakinolide treatment. Since jasplakinolide interrupts actin dynamics, we wanted to investigate whether cell life span would decrease in mutants treated with jasplakinolide. As shown in Figure 2B, jasplakinolide treatment drastically decreased the life span of *tor1Δ, sch9Δ*, and *ras2Δ* mutants, but not the wild type cells. On day 11, without treatment, *tor1Δ, sch9Δ*, and *ras2Δ* mutant viability were 62%, 60%, and 64%, respectively. However, with jasplakinolide treatment, the viability of these mutants decreased to 2%, 8%, and 7%, respectively. These data suggest that a dynamic actin structure is required for longevity of *tor1Δ, sch9Δ*, and *ras2Δ* mutants.

### The deletion of *SLA1* in *tor1Δ*, *sch9Δ*, and *ras2Δ* mutants inhibits actin dynamics and life span

**Figure 3:**
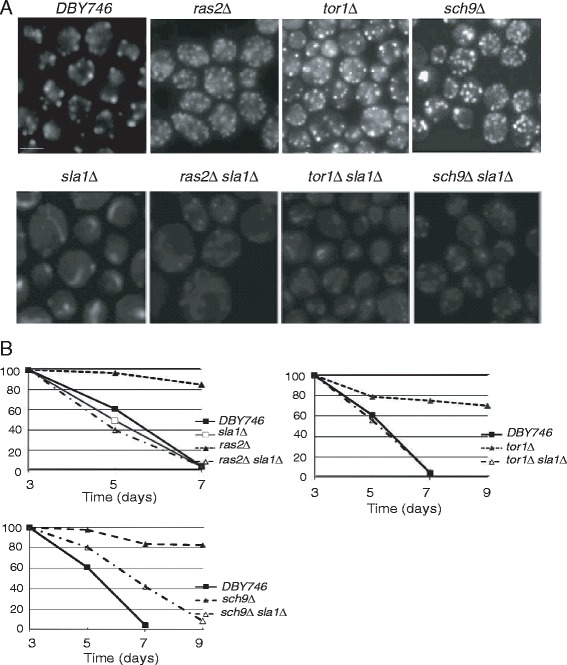
SLA1 is required for actin dynamics and chronological life span in DBY746, *tor1*, *sch9Δ*, and *ras2Δ* cells. **(A)** *tor1Δ sla1Δ* (LY113), *sch9Δ sla1Δ* (LY114), and *ras2Δ sla1Δ* (LY115) showed less actin dynamics than *tor1Δ* (LY101), *sch9Δ* (LY102), and *ras2Δ* (LY103) single mutants. Cells were fixed for 1 h by adding 37% formaldehyde solution directly to growth medium to final concentration of 3.7%. After washing with 1× PBS solution for three times, cells were stained with phalloidin-TRITC in the dark at room temperature for 1 h and images were taken by fluorescent microscope. Scale bar, 5 *μ*m. **(B)** *tor1Δsla1Δ* (LY113), *sch9Δsla1Δ* (LY114), and *ras2Δsla1Δ* (LY115) double mutants have decreased life spans compared to single mutants. Sla1 is associated with actin patches and cables, which organizes the yeast actin cytoskeleton.

Sla1 is associated with actin patches and cables, the two main forms of actin structures. It was reported that Sla1 is required for dynamic actin organization [13]. To further confirm the role of actin dynamics in the longevity of *tor1Δ*, *sch9Δ*, and *ras2Δ* mutants, *SLA1* was deleted in these mutants. As shown in Figure 3A, upon deletion of *SLA1*, actin dynamics was drastically inhibited in both wild type and mutant cells. We also measured the life span of single mutant *tor1Δ*, *sch9Δ*, and *ras2Δ* cells and double mutant *tor1Δ sla1∆*, *sch9Δ sla1∆* and *ras2Δ sla1∆* cells. As expected, Sla1 is required for longevity of *tor1Δ*, *sch9Δ*, and *ras2Δ* (Figure 3B). Taken together, actin dynamics controls the longevity of *tor1Δ*, *sch9Δ*, and *ras2Δ* cells.

### The actin cytoskeleton dynamics in *sch9Δ* mutant cells depends on Rim15 and Msn2/4, while it relies on Rim15 in *tor1∆* and *ras2∆* mutants

**Figure 4:**
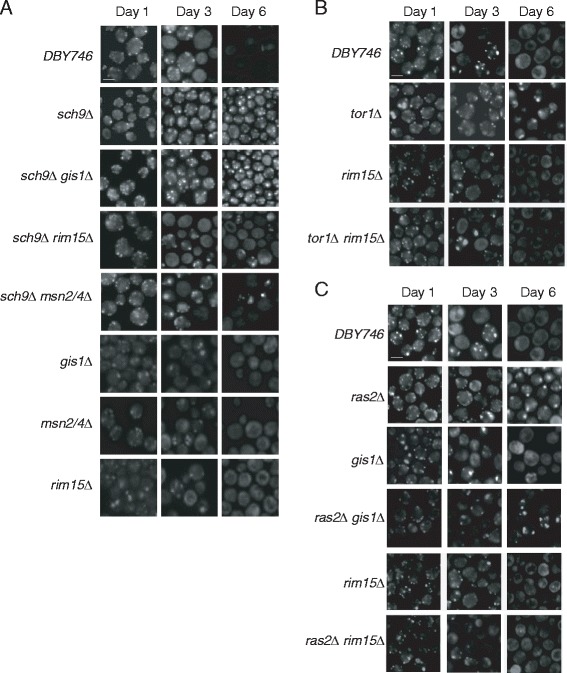
The dynamic actin pattern in *sch9Δ*, *tor1Δ*, and *ras2Δ* cells relies on different effectors. The actin cytoskeleton in *sch9Δ* depends on the RIM15 and MSN2/4 transcription factors, not GIS1 **(A)**, while *tor1Δ* and *ras2Δ* depend on the RIM15 **(B, C)**. Wild type and mutant strains were grown in aging medium. At indicated time points, cells were removed either for testing of cell viability or for fluorescence microscopy. Cells were fixed for 1 h by adding 37% formaldehyde solution directly to the growth medium to a final concentration of 3.7%. After washing with 1× PBS solution for three times, cells were stained with phalloidin-TRITC in the dark at room temperature for 1 h and images were taken by fluorescent microscope. Scale bar, 5 *μ*m.

RIM15, GIS1, and MSN2/4 are effectors of Tor1, Sch9, and Ras2, which are also shown to regulate life span. Previous studies showed that GIS1, RIM15, and MSN2/4 are required for the longevity of *sch9∆* mutants [12], while RIM15 is required for the longevity of *tor1∆,*and Rim15 and Gis1 is required for the longevity of *ras2∆* mutant. Because actin dynamics controls the life span of *tor1Δ*, *sch9Δ*, and *ras2Δ* mutants, we speculate that their effectors may also regulate the actin dynamics in these mutants. To test this hypothesis, we mutated RIM15, GIS1, or MSN2/4 in *sch9Δ*, RIM15 or GIS1 in *ras2∆,* and RIM15 in *tor1∆* mutant. The actin dynamics in these cells were then examined by fluorescent microscopy. As shown in Figure 4A, at day 6, the actin dynamics decreased drastically in the wild type, *sch9∆rim15∆*, and *sch9∆msn2/4∆* cells. However, there was no difference between *sch9∆* and *sch9∆gis1∆* cells. These data indicate RIM15 and MSN2/4, but not GIS1 is required for actin dynamics in *sch9∆* cells. Actin organization was also examined in wild type, *tor1Δ*, *tor1Δrim15∆*, *ras2Δ*, *gis1∆*, *ras2∆gis1Δ* and *ras2Δrim15∆* cells. As shown in Figure 4B and C, the actin structure is dynamic in *tor1Δ, ras2Δ* and *ras2∆gis1Δ*, but not in wild type and *gis1Δ* cells. However, the actin was not dynamic in *tor1Δrim15∆* and *ras2Δrim15∆* double mutants. These data suggests that actin cytoskeleton dynamics in *sch9Δ* cells depends on its effectors RIM15 and MSN2/4, and in *tor1∆* and *ras2∆*, depends on RIM15.

## Discussion

A recent study showed that *Tor1*, *Sch9*, and *Ras2* can regulate carbon source substitution and control calorie restriction in life span extension [17]. Although actin has long been proposed to play an important role in aging [13], whether actin dynamics is involved in the increased life span of *tor1∆*, *sch9Δ*, and *ras2∆* mutants has never been examined. To answer this question, we examined actin organization in the wild type, *tor1∆*, *sch9Δ*, and *ras2∆* mutants by fluorescence microscopy. Consistent with previous reports, the actin structure is dynamic in these longevity mutants but not wild type cells. Numerous bright small separate dots around the cell were found in *tor1∆*, *sch9Δ*, and *ras2∆* mutant cells. To the contrary, actin in the DBY746 wild type looks like a large aggregate clump after day 3 or 5 cultures. The aggregates were shown to be extremely stable structures and less dynamic [16], but the separate smaller dots were not. To further confirm the role of actin dynamics in the aging process, the actin structure was destroyed either by jasplakinolide treatment or through deletion of the *SLA1* gene. Our data indicate that actin dynamics is required for the increased life span of the *sch9Δ*, *tor1∆*, and *ras2∆* mutants. Because RIM15, GIS1, and MSN2/4 are effectors of *Tor1*, *Sch9*, or *Ras2*, which are also shown to regulate life span, we speculate that some of them may be required for maintenance of the dynamic actin pattern in *tor1∆*, *sch9Δ*, and *ras2∆* mutants. The fact that the actin cytoskeleton dynamics in *sch9Δ* depends on RIM15 and MSN2/4 but not GIS1, and in *tor1∆* and *ras2∆*, depends on RIM15, suggests that these effectors also control actin dynamics in *sch9Δ*, *tor1∆* and *ras2∆* mutants. Our study suggested that dynamic actin pattern is required for longevity of *sch9Δ*, *tor1∆* and *ras2∆* mutants. These results will help us to further understand the role of actin in aging.

The mechanism that these mutants regulate actin dynamics, however, is still unclear. In budding yeast, Tor1 functions upstream of the Rho1-Pkc1 pathway, which was shown to regulates actin dynamics [18]. Ho and Bretscher demonstrated that deletion of RAS2 results in loss of Cdc42p and F-actin polarity [19], and it was suggested that Ras2 regulates actin cytoskeleton dynamics via the actin regulatory factor Srv2 [20]. Although there is no direct evidence shows that Sch9 regulates actin dynamics, recent study indicated that Sch9 is a key regulator of sphingolipid metabolism [21], which was shown to be able to regulate actin cytoskeleton through Ypk1 and Pkc1 [22]. These data suggest that Sch9 may indirectly controls actin dynamics. In addition, the effectors of Rim15, Gis1, and Msn2/4 may also mediate actin dynamic pattern during aging. Further investigation is required to answer these questions.

### Experimental procedures

#### Yeast strains and media

Standard methods were used for yeast genetic manipulations [23]. All strains used in this study are listed in Table 1. The deletion mutants were created according to the procedure described previously [24] and confirmed by sequencing.

**Table 1:** **Yeast strains used in this study**

**Name**	**Genotype**	**Reference**
DBY746	MATα *leu2-3 112 his3∆1 trp1-289 ura3-52 GAL+*	[11]
LY101	MATα *leu2-3 112 his3∆1 trp1-289 ura3-52 tor1::KanMX GAL+*	This study
LY102	MATα *leu2-3 112 his3∆1 trp1-289 ura3-52 sch9::KanMX GAL+*	This study
LY103	MATα *leu2-3 112 his3∆1 trp1-289 ura3-52 ras2::KanMX GAL+*	This study
LY104	MATα *leu2-3 112 his3∆1 trp1-289 ura3-52 tor1::KanMX rim15::TRP1 GAL+*	This study
LY107	MATα *leu2-3 112 his3∆1 trp1-289 ura3-52 sch9::KanMX rim15::TRP1 GAL+*	This study
LY108	MATα *leu2-3 112 his3∆1 trp1-289 ura3-52 sch9::KanMX gis1::HIS3 GAL+*	This study
LY109	MATα *leu2-3 112 his3∆1 trp1-289 ura3-52 sch9::KanMX msn2::HIS3 msn4::LEU2 GAL+*	This study
LY110	MATα *leu2-3 112 his3∆1 trp1-289 ura3-52 ras2::KanMX rim15::TRP1 GAL+*	This study
LY111	MATα *leu2-3 112 his3∆1 trp1-289 ura3-52 ras2::KanMX gis1::HIS3 GAL+*	This study
LY113	MATα *leu2-3 112 his3∆1 trp1-289 ura3-52 tor1::KanMX sla1::TRP1 GAL+*	This study
LY114	MATα *leu2-3 112 his3∆1 trp1-289 ura3-52 sch9::KanMX sla1::TRP1 GAL+*	This study
LY115	MATα *leu2-3 112 his3∆1 trp1-289 ura3-52 ras2::KanMX sla1::TRP1 GAL+*	This study

To avoid possible effects due to auxotrophic deficiencies of the strains, the liquid synthetic complete dextrose (SCD) medium was supplemented with a 4-fold excess of tryptophan, leucine, uracil, and histidine (17) and 2% dextrose was used as a carbon source. This, we called “aging medium.” For YPD plates, 19 g L^−1^ bacto-yeast extract, 20 g L^−1^ bacto-peptone, and 20 g L^−1^ bacto-agar (Becton, Dickenson and Company) were dissolved in water and autoclaved and dextrose was added to a final concentration of 2%. YPE and YPG plates were made in the same way as YPD plates, except that 3% ethanol or 3% glycerol was used as a carbon source instead of dextrose.

#### Chronological life span assay

The chronological life span was measured according to the previous report [25]. Briefly, a small amount of yeast strain from the frozen (−80°C) sample was streaked onto an YPD plate and incubated at 30°C for 2–3 days. Five to six colonies were inoculated into 2 ml YPD liquid medium to make the saturated overnight culture (SONC). The fresh SONC was diluted in 25 ml of liquid medium to an initial density of ~1 × 10^6^ cells mL^−1^ (A_600_ = 0.1). The flasks were maintained at 30°C at 220 rpm in the air bath shaker. The initial time point began at day 0, the next day was day 1, and so on. At day 3, the cells reached the stationary phase and stopped dividing, at which point the cells were deemed to be 100% viable. To determine the number of viable cells, 100-*μ*l aliquots were removed from each culture and diluted serially to reach a 1:10^5^ dilution in sterile distilled water. Two dilutions per culture were used routinely, and 100 *μ*l of the dilution was plated onto YPD plates in duplicate. After incubation for 2–3 days, the number of colonies was counted, and the viability at this time point was calculated by comparing the numbers to day 0.

#### Microscopy

Phalloidin-TRITC (Sigma, P1951) stain was performed as previously reported [26]. Cells were fixed for 1 h by adding 37% formaldehyde solution directly to growth medium to a final concentration of 3.7%. After washing with 1× PBS solution three times, cells were stained with phalloidin-TRITC in the dark at room temperature for 1 h. A Nikon Eclipse E600 fluorescence microscope equipped with a Plan Apo 100× 1.4 oil immersion objective was used for fluorescence microscopy. The images were captured with a SPOT RT 9.0 onochrome-6 camera using the MetaMorph (version 6.3.0) acquisition software. The whole cell reconstruction was gained by confocal microscopy (Leica TSC SP2/AOBS). The thickness of the Z-sections is 400 nm. Images were processed by Adobe Photoshop (version 7.0).
